# Cases of Wounded and Diseased Arteries

**Published:** 1827-04

**Authors:** B. Travers

**Affiliations:** St. Thomas's Hospital


### WOUNDED ARTERIES.

*Cases of Wounded and Diseased Arteries, treated principally at* ST. THOMAS'S HOSPITAL, *by* B. TRAVERS, Esq. F.R.S.

(Continued from page 236.)

CASE IV. *Ligature of the Carotid for a Fungoid Tumor of the Cheek, attended by Arterial Hemorrhage.*

John Mansfill, admitted into St. Thomas's Hospital, December 8th, 1814, was the subject of a tumor, which, according to his statement, had appeared six weeks before his admission, after a violent fit of coughing. It was situated upon the top of the right cheek, extending from the infraorbital hole outward beyond the external canthus of the right eye, and downward below the inferior angle of the os malæ; it encroached on the orbit, and pressed slightly against the eyeball. He complained of a slight pain occasionally shooting from the tumor towards the ear; the swelling, and a sense of throbbing, were augmented on lying down in bed, and in coughing. He had a troublesome cough, attended with some morning expectoration.

26th February.—Mr. HENRY CLINE made an incision along the edge of the orbit, extending from the outer canthus to the infraorbital hole. He could discover no vessel supplying the tumor from within the orbit; and, after a dissection of some length, in which nearly the upper half of the tumor was removed, the probe passed, as was supposed, into the cavity of the antrum. The bleeding was profuse, but was checked by compresses. Three days afterwards, an erysipelatous swelling of the face ensued, attended with fever and delirium. To this succeeded considerable pulmonary congestion and dyspnœa, which was removed by bleeding and blisters. The portion of tumor removed was of firm consistence; that which remained gradually increased: the skin covering it was thin, and of a livid colour; the pulsation strong, and continued pressure very painful. The incision did not heal, but was filled by a fungous granulation. In this state he became my patient.

On the night of the 12th of April, an arterial bleeding took place from the fungus, to the amount of three pints. The hemorrhage was arrested by continued pressure upon the carotid on the same side, and compresses applied to the tumor.

On the 13th, at twelve o'clock, advised and assisted by Mr. H. Cline, I tied the common carotid. The operation, which was somewhat tedious, owing to the fulness and shortness of his neck, was satisfactorily completed; the artery alone included in a ligature of twine, and the wound dressed with adhesive plaster.

Ten P.M.—Pretty easy since the operation; pulse 108.

14th, nine A.M.—Pulse 104; passed a good night; complains of a little pain on the opposite side of the head. Takes five drops of laudanum in fever mixture every fourth hour. This was directed to quiet his cough.

15th, nine A.M.—Was relieved by an enema thrown up last night, and had slept well. At seven this morning, took a table-spoonful of castor-oil. Pulse 100; some pain in the neck. Bread poultice applied over the straps. Slight subsultus in the fingers; breathing slightly quickened, and some thirst. The opium was discontinued.

Seven P.M.—Much the same. Complains that the right side of his head feels dead. Oil repeated.

16th, ten A.M.—Slept pretty well. Has been much relieved by four copious motions. Pulse 105; subsultus almost gone; complains of drumming in the head. Wound dressed; healed above and below the ligature. Poultice continued over the straps. Tumor appears more flaccid.

Seven P.M.—Pulse 110; subsultus increased, but not considerably. Has complained this afternoon of pain in his chest, and in the shoulder adjoining the wound; also under the right ear, and at the back of the head. He has now a sense of drumming or thumping on the opposite side of the head. A poultice has been applied to the tumor since the operation.

17th, half-past one P.M.—Pulse ninety-three; little sleep last night. Has had relief three times from a dose of castor-oil taken this morning. The spasms, pain, and uneasy sensations, which prevented his sleeping last night, have all left him.

18th, two P.M.—Pulse 110; considerable febrile irritation; restlessness. A very obscure pulsation is perceived by pressure of the finger upon the tumor. Takes sago boiled in milk, and seems to relish it.

19th, one P.M.—Had a bad night, being much disturbed by his cough, from which he has never been free since he entered the hospital, and has habitually taken the lohoch.* Expectorates much in the fore part of the day. Pulse ninety-five; makes no complaint of pain. Has been again relieved by the operation of castor-oil taken early this morning. A slight difficulty in swallowing, which he has had since the operation, is relieved.

20th, one P.M.—Slept better last night, in consequence of an opiate taken at bedtime. Pulse 100; has little or no pain. The poultice changed since yesterday for simple dressing.

21st, twelve M.—Pulse ninety-two; bowels open.

22d, half-past one P.M.—Pulse ninety-five; slept indifferently last night, and the night before; bowels open; wound healed, except the orifice for the ligature. Has had occasional pain at the back of his head since yesterday.

23d, ten A.M.—Cough disturbed him much last night. Pulse ninety; swallows well; has no pain, except in coughing.

24th.—Was attacked with rigors yesterday, at two P.M., which continued two hours. Ordered an ammonia draught, with twenty drops of tincture of opium. This was followed by a sound sleep and copious perspiration. At three this morning, had another attack of rigor, which continued for half an hour.

* The name of a cough linctus.

Half-past four P.M.—Pulse eighty-one; perspires profusely.

25th, twelve M.—A slight return of rigor last night; took an opiate, and slept well. Pulse 142; pain at the right side and back of his head; subsultus tendinum. Ordered thirty drops of tincture of hyoscyamus in camphor julep every six hours.

26th, twelve M.—Pulse 120. Had a shivering fit an hour ago, which lasted half an hour. Still complains of pain in the back of the head. Castor-oil, taken early this morning, has had no effect. Ordered to be repeated.

27th.—Bowels freely relieved last night. Pulse 110, feeble. Slightly delirious, and has been light-headed at intervals during the last twenty-four hours. Slight oozing of blood from the wound in the neck during the night.

28th, twelve A.M.—Had four loose motions during the night. Pulse 132; delirious; much subsultus tendinum. The tumor is shrunk.

Ten P.M.—Pulse 146; comatose; breathing heavy and difficult. The ligature came away this morning.

29th, one P.M.—Dyspnœa increased; countenance pallid; pulse intermittent, and could not be counted. About two table-spoonfuls of blood issued from the wound at the moment of his death, which took place during the visit.

*Dissection,* 30th April.—*Thorax:* The lungs were much loaded, but not altered in structure.

*Abdomen:* The liver and pancreas had a morbid hardness. The right kidney presented a fungous tumor upon its superior surface, circumscribed, elevated from the cortical surface, resembling on section the tumor in the face, white and hard. There was an hydatid cyst attached to this kidney, and, upon cutting it open, the tubular substance was found to be affected with the fungus, and the ureter was enlarged. The opposite kidney was wasted, and the infundibula formed into pouches, containing some calcareous matter of a brown colour. The tubular structure was nearly obliterated. At the bottom of this kidney was also a large hydatid cyst.

*Head:* A considerable quantity of water was effused between the dura mater and tunica arachnoides. There was no other morbid appearance.

Upon examination of the tumor, it was found that it had no communication with the antrum or orbit, but occupied the whole space between them and the integument. The bone was absorbed at the root of the zygoma, but not considerably. Upon section of this tumor, and that formed in the kidney, they were found to correspond exactly in appearance. Each had an investing cyst, which was filled with a soft medullary substance, in which no intersecting membrane could be observed.

*Carotid:* The artery was divided, and the extremities separated by an interval of three lines. Some injection thrown into the opposite carotid had found its way into the upper portion, insinuating itself by the side of the clot, and filling up the space not occupied by it. The upper clot was unadhering to the internal coat, an inch and a half long, bounded by the superior thyroid artery, about half an inch short of the division. The tunics were completely severed by ulceration, but the internal tunic, separated from the middle and outer, projected above the mouth of the inferior portion of the artery, having a thin ragged appearance, about two lines in length. The inferior clot was an inch in length, not completely filling the tube, and adhering only at its superior extremity to the internal tunic, where it was closely embraced by it. The base of the clot was on the same plane with the round ulcerated edge of the external tunics, and above it the ragged portion of the cuticular coat projected as before described. There was no sign of a healthy adhesive process, nor any contraction of the ends of the vessel. The interior parietes of the wound had an ill-conditioned aspect, being destitute of lymph, though the wound in the integument was healed.

*Remarks.*—The appearances on dissection seem sufficiently to explain the state of constitutional irritation which proved fatal to this man; and to the process which would have been essential, had his life been prolonged, to secure him against a return of hemorrhage. Other cases have afforded evidence of the imperfect state of security in which the ligature of the carotid or subclavian has quitted the artery, by alarming returns of arterial hemorrhage, at intervals even of many weeks. An interesting example of this fact occurred lately in the practice of my colleague Mr. Green, the particulars of which will shortly be communicated to the public. The disposition to early healing of the skin-wound in these cases is apt to deceive us as to the state of the parts beneath. This should not be encouraged. A soft simple dressing of such wounds is preferable to uniting them by adhesive straps, which irritate, and, by agglutinating the edges, favour the confinement of matter.

The operations of tying the carotid and subclavian are not now so formidable as they were twelve years ago, when this case occurred; when the first had been practised with success in very few instances, and the second, although three or four times attempted, had not yet presented a successful result. The demonstrable progress of surgical science in this department within so limited a period of its history, is a truly gratifying reflection, and it would serve as an admirable incentive, as well as guide, to the zeal and industry of its votaries, if an impartial critical inquiry were occasionally carried into all its departments, which should place the neglected in contrast with the cultivated spots.

"Ita res accendent lumina rebus."

The carotid has been tied, not only for wounds and aneurisms, direct and anastomotic, nævi and bleeding fungi of the face, and active hemorrhage following the extraction of a tooth, but as an antecedent measure to the removal of a portion of the lower jaw, in the disease termed osteo-sarcoma; in one case at the interval of a day, and in another immediately preliminary to the operation. This was American surgery, which has presented us with other instances of an unexampled but successful boldness. In the cases in which the ultimate object of the ligature of the carotid has failed of accomplishment, it has seldom aggravated the condition of the patient; and in a large proportion it has effected all that was expected of it. But the fact recently published by Mr. WARDROP, if borne out by similar results, may be ranked as next in value to the original discovery of the practicability and safety of including the carotid in a ligature, and its efficacy for the cure of aneurism. And the proposition has the higher merit, as it is a new application of the same principle as that upon which we explain the cure of aneurism in ordinary cases, derived, as he informs us, from reflection upon that process.* At present, however, we are not authorised to expect that a ligature beyond the sac would be applicable in any case in which a permanent branch arose from the sac, except upon conjecture that such branch had become obliterated, which is far from improbable; or, secondly, in which the ligature must necessarily be contiguous to a considerable branch, as, for example, above the profunda femoris or epigastric artery.

These conditions seem almost to limit the application of this mode of operating to the common carotid. But it is probable that the utility of the ligature is not confined to the cases in which it positively intercepts all current: blood in motion will coagulate, though not so quickly as when at rest. Mr. Hewson, indeed, says, "I have found that it coagulates as soon when kept warm and when agitated, as it does when suffered to rest and to cool." It is fair to add, however, that he attributed its coagulation to rest "in those true aneurisms which are attended with a pouch." "For, in such enlargements, a part of the blood is without motion, which will congeal when at rest and in contact with the sac, and thus one layer may be formed, &c.; and thence, probably, is the origin of those laminated coagula met with in such sacs."†

Following out the principle above hinted at, future emergencies will develop what degree of restraint or remora of the

* Medico-Chirurgical Transactions, vol. xiii. part i.
† Experimental Inquiry into the Properties of the Blood, p. 17, 26.

current is sufficient to bring about the eventual obliteration of the sac. The subsequent morbid changes upon the integument of the sac,—the suppuration of the sac itself, and the expulsion of its contents, are unimportant consequences as regards the disease, and occasionally seen after the operation as hitherto practised for aneurisms of the lower limbs, in unfavourable states of health or constitution.

I annex an example from my own practice. If an aneurismal sac could be converted into an abscess, properly so called, there would be little danger of hemorrhage.

Case V. *Popliteal Aneurism, with Gangrene of the Integument, in which the Sac suppurated after the Operation, and superficial Gangrene occurred in several parts of the Leg.*

C. F. Hiellstrom, æt. thirty, a tall Swedish sailor, of a spare, irritable habit, was admitted, February 20th, 1823, with a very painful tumor filling the entire ham, and pulsating violently. First noticed it a fortnight ago, since which time it has increased so much as to prevent even a partial flexion of the knee.

Feb. 24th.—The tumor had increased, and near its centre appeared a discoloured spot, the size of a half-crown piece. The femoral artery was tied with a single ligature. The discoloration continued to extend; and, on the 28th February, a slough, as large as a sixpenny piece, separated, when about one ounce of grumous blood escaped. Further discharge was prevented by the application of a pledget of lint.

March 1st.—Sp. Tereb.; Ung. Simpl. āā p. æq. M. f. ung. vul. poplitis app.

3d.—No adhesion of the edges of the incision had taken place, and the wound had an unhealthy appearance.

Sumat Cerev. ℔ij. indies.—℞. Dec. Cinchonæ ℥jss.; Tr. Ejusd. Co. ʒj.; Pulv. Ejusd. ʒj. M. ter die sumend.—Poplit. adhib. Catap. Spum. Cerev.

On the 8th, a blush of erysipelatous inflammation appeared about the knee.

Catapl. cum Lot. alb.—Adde sing. haust. Tr. Opii m. v.—Sumat Vini rubri ℥iv. indies.

14th.—The ligature separated. From the aneurismal sac there is a copious discharge of purulent matter, mixed with grumous blood. Apparently from pressure by the posture in which the leg lies, a discoloured patch has formed on its outer side, which on the following day became gangrenous.

Applic^r Cataplasma Cerev.

The wound of the thigh discharged copiously for some time; but, by careful rolling, the sinuses were obliterated, when the man's health improved.

25th.—Sumat Vini rubri ℥viij. indies. — very good

April 9th.—The wound from the operation had quite healed; the ulcers on the leg and in the ham were clean and granulating; the sac much diminished.

May 1st.—A gangrenous sore appeared on the outer edge of the foot.

Applic^r Lotio Acid. Nitr. dil. (gtt. vj. ad Aquæ ℥ j).

25th.—The sore on the leg, which was nearly healed, again attacked by gangrene; that on the foot is deep and foul.

Cataplasma Cerev.

29th.—Three considerable gangrenous spots, which soon became sores, on the outer side of the foot, with much surrounding dusky redness.

Lint, dipped in the Nitric Acid Lotion, was applied beneath the yeast poultice.

June 20th.—The sores had all filled up with healthy granulations.—Empl. Sapon.

September 10th.—Discharged quite well.

CASE VI. *Subclavian Aneurism, in which the Operation was followed by Inflammation of the Right Pleural Sac, which proved fatal.*

Wm. Cottrell, æt. seventy-three, a countryman, admitted Jan. 9th, 1823, with a very painful, strongly pulsating tumor, as large as a swan's egg, protruding the pectoral muscle, and extending to the clavicle. He first perceived a swelling on the right breast about three months ago, and it has since been gradually increasing. Having for some previous days taken aperient medicine, the subclavian artery was tied above the clavicle on Jan. 17th. The sac having given way in the act of passing the needle, much blood was lost, and, although the artery was perfectly secured, the ligature did not command the bleeding, and it was found necessary to introduce a sponge tent into the wound, by which the hemorrhage was controlled. In the evening, the cough, to which he had long been subject, became very troublesome.

Sumat Linctus Papav.

The following morning, Jan. 18th, he complained of uneasiness down the spine; pulse 100; little pain in the wound and arm; had slept two hours.—Sumat Mist. Effervesc.

Eleven P.M.—Pulse 125, and bounding; respiration oppressed; pain in the back increased.

V.S. ad ℥xvj.—Magnesiæ Sulph. ʒj. quartis horis sumend.

—The blood had a thick buffy coat.

Jan. 19th.—Pulse 117; breathing oppressed. For some hours has had frequent spasmodic action of the diaphragm.

The wound dressed simply.

Nine P.M.—The arm is warm, but benumbed; still complains of pain extending down the spine; breathing very troublesome; great anxiety; pulse 130. Has had two stools.

℞. Liq. Antim. Tart., Tinct. Hyoscyami, āā gtt. xxx. ex Aquæ Menth. ℥jss. M. quartis horis sumend.—Applic^r Vulner. Cataplasma Lini.

20th.—The wound dressed, and the sponge removed: the wound has a healthy appearance. In the course of the day, the breathing became stertorous, and painful on inspiration; the complaints of pain in the spine were more frequent; the anxiety of countenance was augmented; and the pulse sunk gradually, till at eleven P.M. he died.

*Dissection.*—On examination of the body, the structure of the lungs was found healthy, but the right sac of the pleura was much inflamed, and it contained about twenty ounces of serum, with floating flakes of coagulable lymph. The aneurismal sac was nearly empty. No adhesion had taken place in the wound, in which a small quantity of pus had been secreted. On minute examination, it was ascertained that the ligatures were firmly seated on the artery at the root of the sac, and adjoining the outer margin of the scalenus. The sac had a pouch-like enlargement upwards, which closely overlaid the artery on the pectoral side; and this having been penetrated in the passage of the needle, had occasioned the profuse arterial hemorrhage without saltus, which was not arrested by the tightening of the ligature. On this account a piece of sponge tent was placed within the lips of the wound.

*Remarks.*—Could the circumstance above mentioned have been foreseen, I need scarcely observe, the operation would have been regarded as impracticable. The hemorrhage was more terrific and uncontrollable than I ever witnessed. It commenced at the moment above stated, and was concluded to come from a rupture of the sac, being void of pulsation, and not commanded by drawing the ligature tight, and afterwards passing another above the first. The quantity of blood lost was so great, that it was at one time extremely doubtful whether the man would quit the theatre alive. His age, added to this circumstance, was thought to render the repetition of venesection, beyond a single bleeding, inadmissible. This was, perhaps, an error in judgment.—Some gentlemen attributed the inflammation of the pleura to the long exposure of the chest in the reduced state of the circulation; but the unavoidable introduction of a foreign substance into the wound, and the forcible separation and subsequent inflammation of parts thereby occasioned, is a more probable explanation. The thoracic cavity was uninjured in its integrity. I have seen two instances of the sympathetic inflammation of the contiguous pleura, from the amputation of schirrous tumors of the breast, which proved fatal at little more than the same interval of time; and a third from the inflammation, after incision, of the sac of a large chronic abscess, situated beneath the scapular and pectoral muscles.

The pain referred to the cervical spine, and frequent spasmodic affection of the diaphragm, were evidences of the acute irritation of the cervical and phrenic nerves.

(To be continued.)

